# Attention Bias to Pain Words Comes Early and Cognitive Load Matters: Evidence from an ERP Study on Experimental Pain

**DOI:** 10.1155/2021/9940889

**Published:** 2021-10-31

**Authors:** Kangling Wang, Yifei Chen, Shimin Huang, Howe Liu, Wen Wu

**Affiliations:** ^1^Department of Rehabilitation Medicine, Zhujiang Hospital, Southern Medical University, Guangzhou, China; ^2^Physical Therapy, Health Science Center, University of North Texas, Fort Worth, TX, USA

## Abstract

Attention bias (AB) is a common cognitive challenge for patients with pain. In this study, we tested at what stage AB to pain occurs in participants with experimental pain (EP) and tested whether cognitive load interferes with it. We recruited 40 healthy adults aged 18-27 years, and randomized them into control and EP groups. We sprayed the participants in the EP group with 10% capsaicin paste to mimic acute pain and those in the control group with water, accessing both groups' behavioral results and event-related potential data. We found that high-load tasks had longer response times and lower accuracies than low-load tasks did and that different neural processing of words occurred between the groups. The EP group exhibited AB to pain at an early stage with both attentional avoidance (N1 latency) and facilitated attention (P2 amplitude) to pain words. The control group coped with semantic differentiation (N1) at first, followed by pain word discrimination (P2). In addition, AB to pain occurred only in low-load tasks. As the cognitive load multiplied, we did not find AB in the EP group. Therefore, our study adds further evidence for AB to pain, suggesting the implementation of cognitive load in future AB therapy.

## 1. Introduction

Attention bias (AB) refers to different allocations of attention to certain types of stimuli [1]. AB to pain-related materials is a common phenomenon in patients with pain [2], and attention bias modification (ABM) is now a novel treatment for pain. However, ABM has been reported to have contradictory result with analgesic effects [3–5] or no substantive improvements [6]. As such, more information regarding AB is needed.

When does AB to pain occur? One study with healthy persons and patients with anxiety found that AB to negative stimuli occurred in an early stage of information processing [7–9]. However, other studies found that AB to pain was limited and inconsistent. For example, Knost et al. [10] reported that pain-related words activated only a stronger early component N100 in patients with chronic pain, as compared with healthy persons. As compared with neutral words, pain-related words can induce stronger late low waves in patients with chronic pain. Sitges et al. [11] have revealed that for patients with pain, pain-related words can elicit significantly more enhanced positive event-related potential (ERP) amplitudes than pleasant words can, but this phenomenon was not confined to a certain period. Until now, no research has provided clear understanding regarding when AB to pain occurs.

Cognitive load is a potential factor influencing pain perception [12–15]. It is generally believed that when compared with low cognitive tasks, medium-to-high cognitive tasks compete with pain for more attention and thus have more obvious analgesic effects [16–18]. Scholars have further confirmed this finding using functional magnetic resonance (fMRI) showing that complicated tasks can activate or deactivate certain brain areas related to pain [19–21]. However, what will happen in AB with different cognitive loads? If a high cognitive load affects one's AB, then the load factor could be used considerably as a new type of intervention.

Building on the above researches, we suggest that AB to pain probably happens at an early stage for persons with pain, and cognitive load may influence AB. To test our hypothesis, we studied a healthy control group and an experimental pain (EP) group using a working memory paradigm of cognitive tasks, with four types of words interspersed as nonstimulus target interferences. We included high and low cognitive loads and obtained and analyzed behavior results and ERP data between the groups. In this study, we assumed that AB to pain stimuli might occur early in the EP group with a significant difference of early ERP components among stimuli and between groups, and that cognitive load can affect AB in a way that the higher the load is, the less obvious AB is.

## 2. Materials and Methods

### 2.1. Participants

We recruited 40 healthy students (20 men and 20 women; aged 18-27 years) from the University of Southern Medical University. The inclusion criteria were right-handed, fluency in Chinese, and normal or corrected-to-normal vision. The exclusion criteria were having a diagnosis of or receiving treatment for a psychiatric disorder currently or within the past 5 years or regularly taking any psychotropic or analgesic medications. All students gave their written informed consent to participate in the study, which was approved by the Ethics Committee of Zhujiang Hospital, Southern Medical University.

We obtained participant demographic data during the evaluation session before the experiments. We collected scores from the Hospital Anxiety and Depression Scale (HADS) and the State-Trait Anxiety Inventory (STAI) afterwards.

### 2.2. Experimental Protocol

The experiment was designed for 2 groups (control and EP) × 2 cognitive loads (high and low) × 4 interfering words (neutral, positive, negative, and pain). We randomized the participants into EP and control groups. EP participants were sprayed with 10% capsaicin paste (Professional Arts Pharmacy) on the left inner forearm and covered with plastic wrap to mimic a sense of acute pain. The control participants were sprayed with pure water and covered with plastic wrap also. Cognitive load was distinguished by the length of a number string (6 for high, 2 for low). All 400 digits were generated by a random number generator, with 50% in each kind of digits, respectively.

Interfering word types included neutral, positive, negative, and pain. We selected 25 neutral, positive, and negative words, respectively, from the modern Chinese lexicon in the Chinese emotional material database, with the low-level features (i.e., valence, arousal rating, dominance, and familiarity) matched for each type. Sensory pain words (i.e., dull pain) were selected from the Chinese version of the McGill Pain Questionnaire (MPQ).

Because the pain words in the MPQ were not large enough in quantity to form enough stimulus for ERP research, we presented all 25 words in 4 forms with 4 different colors (blue, green, red, and yellow), resulting in 100 words in each word category. Color saturation and brightness were matched to eliminate errors between the colors. Words and cognitive loads were randomly combined.

We used the visual analogue scale (VAS) to score pain values before and during the experiment. If a participant's pain perception was lower than 4/10 on the VAS, we resprayed the pain-causing substance on the left inner forearm to maintain a pain perception of >4/10.

**Figure 1:**
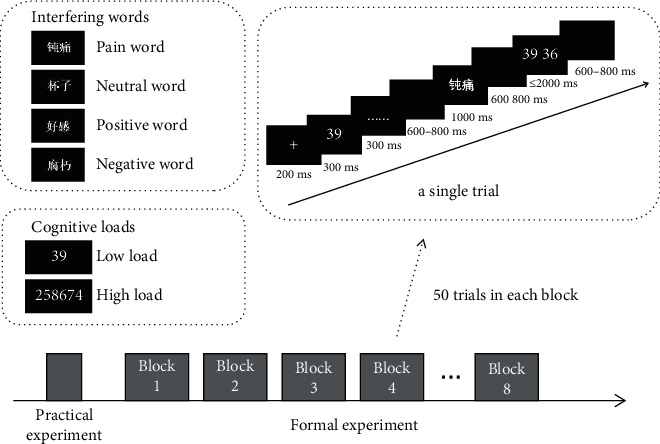
Experiment procedure. Practical experiment was carried out in order to familiarize subjects with the experiment. Formal experiment included 8 blocks with 50 trials in each block, which is shown in the upper right picture. Examples of word and load types are shown in the upper left pictures.

E-Prime version 3.0 software was used for experimental programming. Every presentation of a trial began with a fixation point “+” (200 ms), followed by a sequence of digital loads (300 ms) and a “......” screen to maintain the width of attention (300 ms), an empty screen (600-800 ms), a word interference screen (1,000 ms), a black screen (600-800 ms), and a selection screen(≤2,000 ms), which consisted of 2 of the same load numbers, with only 1 of them having been presented before. On the selection screen, we asked the participants to respond as quickly and correctly as possible if the numbers appearing previously were on the left-hand side (their left index finger pressing the “F” key) or on the right-hand side (their right index finger pressing the “J” key correspondingly). Finally, we used a black screen (600-800 ms) to end the trial (see Figure 1, upper right).

Before the formal experiment, the participants conducted 24 practices to familiarize themselves with the tasks. To obtain more reliable ERP data, more trials should be conducted. All participants conducted 400 trials in the formal experiment, which we divided into 8 blocks with 50 trials per block. We set the interval between two blocks at 2 min. The entire experiment took 40-60 min. Figure 1 shows the experimental procedure.

### 2.3. EEG Recording and Processing

We recorded electroencephalography (EEG) using a 32-channel cap according to the International 10-20 Electrode Placement System (BioSemi). While recording the EEGs online, we used the average value of the bilateral mastoids as a reference and the AFz electrode as the grounding electrode. We made vertical electrooculogram (EOG) recordings using electrodes placed above and below the left eye and recorded horizontal eye movements using electrodes placed over the outer canthus of both eyes. EEG signals were filtered using 0.05-100 Hz, with 512 Hz as the sampling rate. All interelectrode impedances were maintained below 5 k*Ω*.

Because we mainly examined the effects of the interfering words, the interfering word screen was used as the stimulus onset, and epochs of the former 100 ms and later 600 ms were analyzed. We used the 100 ms waveform before 0 point as the baseline. ERP processing was conducted with MTLAB R2013a (RRID:SCR_001622; MATLAB) and EEGLAB 12.0 (RRID:SCR_007292; EEGLAB). After reducing the sampling rate to 500 Hz, we filtered the data through 0.1-40 Hz.

Continuous data were segmented into the epochs mentioned above. First, bad epochs were marked if more than 20% individual electrodes contained artifacts, and files that contained more than 10 bad channels were discarded from further analysis. Additionally, we performed visual inspection trial-by-trial to ensure that trails with large interference and unstable baseline were appropriately rejected. Independent component analysis (ICA) was conducted to eliminate EOG and electromyogram activities after we removed the bad epochs and channels and the interpolation of the electrodes with high noise. Any epochs with voltage values exceeding ±100 *μ*V were rejected accordingly. Only the epochs of the correct responses were averaged. Finally, each condition had enough epochs (at least 30) for average to ensure a variability of the EEG data.

### 2.4. Data and Statistical Analysis

#### 2.4.1. Behavioral Data

We compared the behavioral indexes related to the four types of words. E-Prime 3.0 was used to extract the indexes as response time (RT) and accuracy (AC). Trials with incorrect responses and nonresponses were deleted. We conducted three-way repeated measures analyses of variance (RM-ANOVAs; SPSS version 20.0; IBM) to examine the differences in RT and AC as a function of the interfering word, cognitive load, and group, in which group was chosen as the between-participant factor and cognitive load and interfering word as the within-participant factors.

#### 2.4.2. ERP Data

After examining the grand-averaged waveforms in our study and referring to those in previous studies [7, 22], we set the time windows for the ERP components as follows: N1, 70-170 ms, with a peak at about 120 ms; P2, 150-250 ms, with a peak at about 200 ms; and N3, 250-350 ms, with a peak at about 300 ms. We included nine electrodes in the analysis on the basis of previous findings according to the frontal (F3, Fz, and F4), central (C3, Cz, and C4), and partial (P3, Pz, and P4) sides, as reported elsewhere [22, 23].

Amplitudes and latencies of N1, P2, and N3 were subjected to four-way RM-ANOVAs, with cognitive load, interfering word, and electrode site as the within-participant factors and group as the between-participant factor. Three-way RM-AVOVAs were conducted if any interactive effects occurred, followed by two-way RM-AVOVAs. Simple effect analysis was performed if interactions between any of the variables were significant. Bonferroni adjustments for multiple comparisons were used for post hoc analyses. Probability values were corrected using Greenhouse-Geisser correction for multiple degrees of freedom when violations of the sphericity assumption occurred.

## 3. Results

### 3.1. Descriptive Participant Data

Table 1 lists the basic participant information. Because this study is part of a past study, the participant information was the same as in our previous work [24].

### 3.2. Behavioral Performance

**Figure 2:**
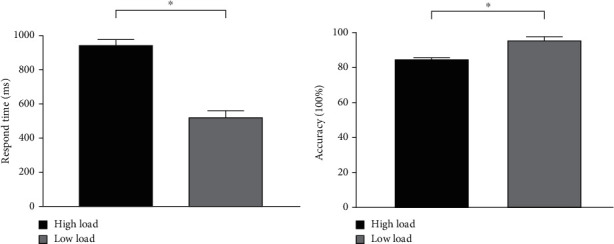
Behavioral results. (a) RT was longer under high cognitive load compared with low cognitive load. (b) AC for low cognitive load was higher compared with that for high cognitive load. ∗ indicates significant difference.

Figures 2(a) and 2(b) show the behavioral results for RT and AC, respectively, for both groups. For RT, a significant interactive effect occurred for cognitive load and interfering word (*F*_(3,114)_ = 3.063, *P* = 0.033). A simple effect showed that for all word types, RT was longer under high load as compared with low load (all *P* < 0.001). A significant main effect occurred for load (*F*_(1, 38)_ = 355.907, *P* < 0.001), with a longer RT under high load (943.637 ± 22.852 ms) as compared to that under low load (533.659 ± 22.852 ms). The main effects for word (*F*_(3,114)_ = 0.563, *P* = 0.457) and group (*F*_(1, 38)_ = 0.074, *P* = 0.787) were not significant. No other interactive effects were found.

For AC, we also found no interactive effects. A significant main effect for load (*F*_(1, 38)_ = 89.933, *P* < 0.001) was found, with a lower AC under high load (85.538% ± 0.830%), as compared with low load (96.673% ± 0.830%). The main effects for word (*F*_(3,114)_ = 2.235, *P* = 0.141) and group (*F*_(1, 38)_ = 2.629, *P* = 0.111) were not significant.

### 3.3. ERP Data

First, we found significant differences in the N1, P2, and N3 amplitudes for the interactive effects in the group, load, word, and electrode site after four-way RM-ANOVAs (N1: *F*_(6,228)_ = 3.097, *P* = 0.028; P2: *F*_(6,228)_ = 5.117, *P* = 0.001; and N3: *F*_(6,228)_ = 5.051, *P* = 0.001). Because data analysis by group would help in more clearly understanding each group's ERP characteristics, we further analyzed the three-way RM-ANOVAs using this method.

### 3.4. Control Group

**Figure 3:**
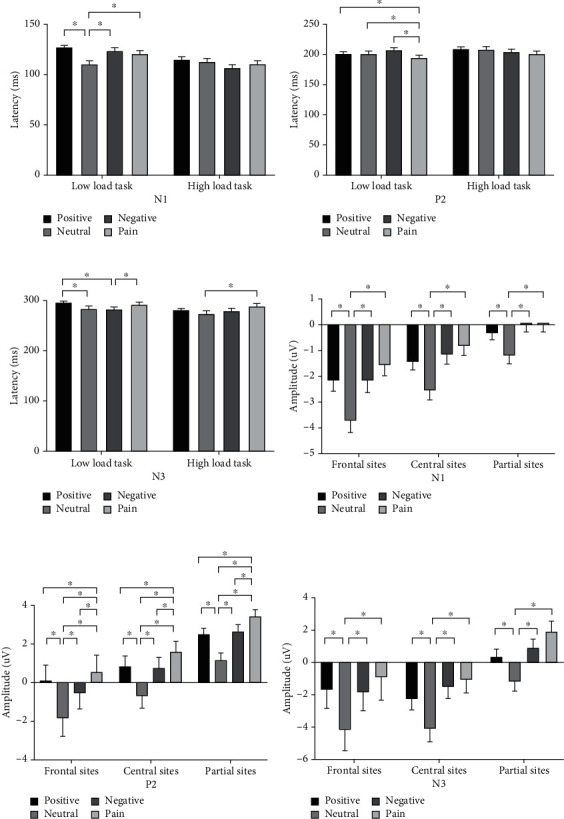
T-bar plots of the ERP components in the control group. Word difference between neutral words and other words was significant in N1 latency and amplitude (a, d). Word difference between pain words and other words was significant in P2 latency and amplitude (b, e). Word difference between neutral words and other words was not significant in N3 latency (c), but significant in N3 amplitude (f). ∗ means *P* < 0.05.

**Figure 4:**
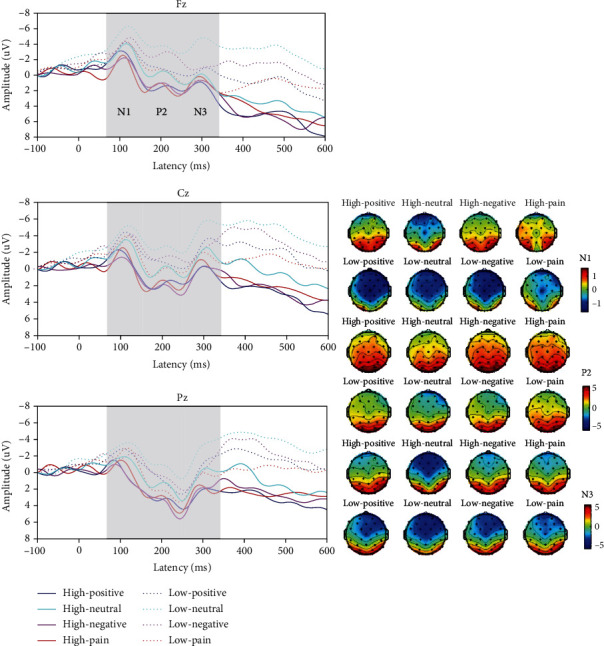
Grand-averaged ERPs and topographic maps in the control group. Left: grand-averaged ERP waves in Fz, Cz, and Pz sites. Right: topographic maps of N1, P2, and N3.

We conducted three-way RM-ANOVAs with load, word, and electrode site as the within-participant factors.  Figure 3 shows the latency (abc) and amplitude (def) results.  Figure 4 shows the grand-averaged ERPs and topographic maps.

#### 3.4.1. N1

For latency, we found a significant interactive effect for load and word (*F*_(3,114)_ = 4.681, *P* < 0.001). In low-load tasks, a significant difference occurred in word (*F*_(3,114)_ = 13.249, *P* < 0.001), mainly between neutral words and other words, including between neutral and positive words (110.441 ± 3.073 ms vs. 126.848 ± 2.391 ms, *P* < 0.001), between neutral and negative words (110.441 ± 3.073 ms vs. 123.994 ± 2.673 ms, *P* < 0.001), and between neutral and pain words (110.441 ± 3.073 ms vs. 120.678 ± 2.994 ms, *P* = 0.019). In high-load tasks, a significant difference occurred among words (*F*_(3,114)_ = 3.005, *P* = 0.030), but only between positive and negative words (114.75 ± 2.86 ms vs. 107.06 ± 2.76 ms, *P* = 0.007).  Figure 3(a) shows these results.

All words had significant differences between high- and low-load tasks (positive: 114.748 ± 2.860 ms vs. 126.848 ± 2.391 ms, *P* < 0.001; negative: 107.057 ± 2.757 ms vs. 123.994 ± 2.673 ms, *P* < 0.001; pain: 110.441 ± 3.073 ms vs. 120.678 ± 2.994 ms, *P* = 0.001), except for neural words (112.868 ± 2.894 ms vs. 110.441 ± 3.073 ms, *P* = 0.416). A significant main effect occurred for load (*F*_(1, 38)_ = 12.670, *P* = 0.001), with a shorter latency in high-load than in low-load tasks (111.278 ± 2.376 ms vs. 120.490 ± 2.217 ms). No other main effect or interactive effects were found.

For amplitude, we found significant interactive effects for load, word, and electrode site (*F*_(6,228)_ = 7.072, *P* < 0.001). The interactive effects for word and electrode site were significant in both high- (*F*_(6,228)_ = 5.541, *P* = 0.001) and low-load tasks (*F*_(6,228)_ = 5.212, *P* = 0.002). A significant difference occurred between neutral and other words in the frontal (*F*_(3,114)_ = 15.593, *P* < 0.001), central (*F*_(3,114)_ = 17.865, *P* < 0.001), and partial sites (*F*_(3,114)_ = 13.528, *P* < 0.001).  Figure 3(d) shows these results. We also found significant differences in each word type, with the frontal sites having the largest negative amplitudes, followed by the central sites and partial sites (positive: *F*_(2, 76)_ = 29.975, *P* < 0.001; neutral: *F*_(2, 76)_ = 57.774, *P* < 0.001; negative: *F*_(2, 76)_ = 39.386, *P* < 0.001; and pain: *F*_(2, 76)_ = 17.013, *P* < 0.001).

#### 3.4.2. P2

For latency, we found a significant interactive effect for load and word (*F*_(3,114)_ = 11.657, *P* = 0.004). In low-load tasks, a significant difference occurred in words (*F*_(3,114)_ = 8.034, *P* < 0.001), mainly between pain and other words, including between pain and positive words (194.955 ± 3.445 ms vs. 200.630 ± 3.100 ms, *P* = 0.045), between pain and neutral words (194.955 ± 3.445 ms vs. 201.672 ± 3.415 ms, *P* =0.008), and between pain and negative words (194.955 ± 3.445 ms vs. 207.756 ± 3.352 ms, *P* < 0.001). In high-load tasks, no significant difference occurred among words (*F*_(3,114)_ = 2.726, *P* = 0.054).  Figure 3(b) shows these results.

We found a significant difference between high- and low-load tasks for three word categories (positive: *F*_(1, 38)_ = 14.799, *P* < 0.001; neutral: *F*_(1, 38)_ = 7.507, *P* = 0.007; pain: *F*_(1, 38)_ = 13.085, *P* < 0.001), with a longer latency in high-load tasks than in low-load tasks, except for negative words (*F*_(1, 38)_ = 0.555, *P* = 0.458). We found no other interactive effect or main effects.

For amplitude, we found a significant interactive effect for word and electrode site (*F*_(6,228)_ = 73.208, *P* = 0.023). A significant difference occurred between pain and other words and between neutral words, as well as in the frontal (*F*_(3,114)_ = 14.995, *P* < 0.001), central (*F*_(3,114)_ = 22.865, *P* < 0.001), and partial sites (*F*_(3,114)_ = 24.752, *P* < 0.001).  Figure 3(e) shows these results.

Significant differences occurred in each word category, and the partial sites had the largest positive amplitude, followed by the frontal and central sites (positive: *F*_(2, 76)_ = 12.503, *P* < 0.001; neutral: *F*_(2, 76)_ = 18.328, *P* < 0.001; negative: *F*_(2, 76)_ = 28.097, *P* < 0.001; and pain: *F*_(2, 76)_ = 16.451, *P* < 0.001). We found no other interactive or main effects.

#### 3.4.3. N3

For latency, we found a significant interactive effect for load and word (*F*_(3,114)_ = 3.140, *P* = 0.029). In low-load tasks, a significant difference occurred in words (*F*_(3,114)_ = 6.047, *P* = 0.001), including between positive and neutral words (294.424 ± 3.783 ms vs. 284.580 ± 3.753 ms, *P* = 0.016), between positive and negative words (294.424 ± 3.783 ms vs. 282.751 ± 3.638 ms, *P* = 0.006), and between negative and pain words (282.751 ± 3.638 ms vs. 291.860 ± 3.760 ms, *P* = 0.018). In high-load tasks, a significant difference occurred between words (*F*_(3,114)_ = 5.315, *P* = 0.002) but only between neutral and pain words (275.470 ± 3.283 ms vs. 289.844 ± 3.481 ms, *P* = 0.008).  Figure 3(c) shows these results.

We found a significant interactive effect for word and electrode site (*F*_(6,228)_ = 3.782, *P* = 0.005). A significant difference occurred in each word type: the partial sites had the shortest latency, followed by the frontal sites and central sites (positive: *F*_(2, 76)_ = 28.003, *P* < 0.001; neutral: *F*_(2, 76)_ = 16.126, *P* < 0.001; negative: *F*_(2, 76)_ = 20.781, *P* < 0.001; and pain: *F*_(2, 76)_ = 16.258, *P* < 0.001). We found no significant difference between words in the frontal (*F*_(3,114)_ = 1.530, *P* = 0.207), central (*F*_(3,114)_ = 0.046, *P* = 0.831), or partial sites (*F*_(3,114)_ = 0.977, *P* = 0.404). We found no other interactive or main effects.

For amplitude, we found a significant interactive effect for word and electrode site (*F*_(6,228)_ = 2.927, *P* = 0.041), but no significant main effects for word (*F*_(3,114)_ = 2.397, *P* = 0.078) or electrode site (*F*_(2, 76)_ = 3.007, *P* = 0.058). A significant difference was found between neutral and other words in the frontal (*F*_(3,114)_ = 12.521, *P* < 0.001), central (*F*_(3,114)_ = 21.996, *P* < 0.001), and partial sites (*F*_(3,114)_ = 25.277, *P* < 0.001).  Figure 3(f) shows these results. We found no other interactive or main effects.

### 3.5. Experimental Pain Group

**Figure 5:**
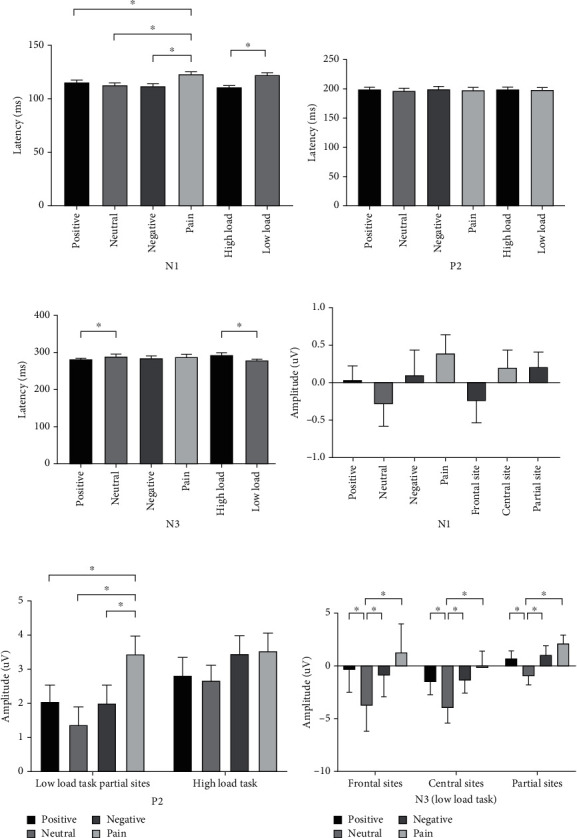
T-bar plots of the ERP components in the experimental pain group. Word difference between pain and other words was significant in N1 latency and P2 amplitude (a, e). Word difference between neutral and other words was significant in N3 amplitude only in low load tasks (f). ∗ means *P* < 0.05.

**Figure 6:**
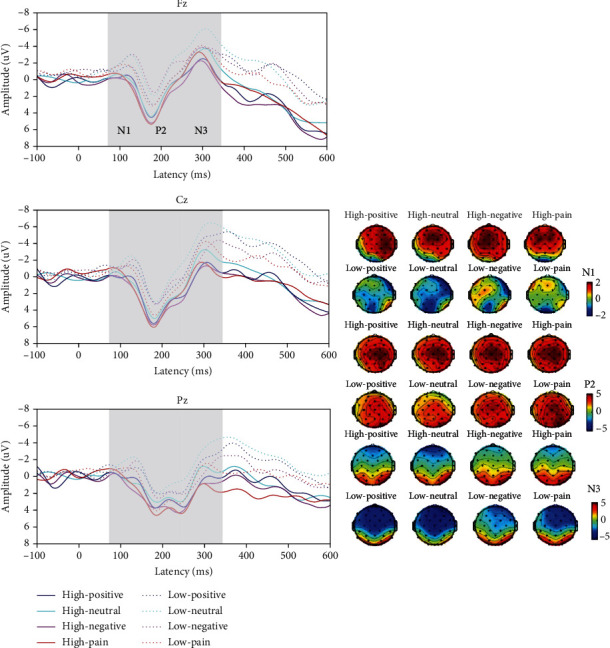
Grand-averaged ERPs and topographic maps in the EP group. Left: grand-averaged ERP waves in Fz, Cz, and Pz sites. Right: topographic maps of N1, P2, and N3.

Figure 5 shows the results for latency (abc) and amplitude (def).  Figure 6 shows the grand-averaged ERPs and topographic maps.

#### 3.5.1. N1

For latency, we found no significant interactive effect for load and word (*F*_(3,114)_ = 3.657, *P* = 0.052) but did find significant main effects for word (*F*_(3,114)_ = 13.736, *P* < 0.001) and load (*F*_(1, 38)_ = 36.688, *P* < 0.001). Pain words had the longest latencies, versus those for the other words (positive: 115.748 ± 1.933 ms; neutral: 113.252 ± 1.922 ms; negative: 112.740 ± 1.810 ms; and pain: 123.755 ± 2.068 ms). High-load tasks (110.368 ± 1.765 ms) had shorter latencies than low-load tasks (122.396 ± 1.876 ms) did.  Figure 5(a) shows these results. We found no other interactive or main effects.

For amplitude, we found no significant interactive effect for word and electrode site (*F*_(6,228)_ = 2.741, *P* = 0.052), nor did we find significant main effects for word (*F*_(3,114)_ = 2.397, *P* = 0.078) or electrode site (*F*_(2, 76)_ = 3.903, *P* = 0.056).  Figure 5(d) shows these results. We found no other interactive or main effects.

#### 3.5.2. P2

For latency, we found a significant interactive effect for word and electrode site (*F*_(6,228)_ = 3.123, *P* = 0.018). No significant difference occurred among words in the frontal (*F*_(3,114)_ = 1.558, *P* = 0.200), central (*F*_(3,114)_ = 0.735, *P* = 0.477), and partial sites (*F*_(3,114)_ = 2.662, *P* = 0.058). However, a significant difference was found among electrode sites in each word category (positive: *F*_(2, 76)_ = 38.667, *P* < 0.001; neutral: *F*_(2, 76)_ = 36.261, *P* < 0.001; negative: *F*_(2, 76)_ = 36.860, *P* < 0.001; and pain: *F*_(2, 76)_ = 30.336, *P* < 0.001), with the shortest latency in the partial sites, followed by the central and frontal sites. We found no significant main effect for load (*F*_(1, 38)_ = 0.779, *P* = 0.383) or word (*F*_(3,114)_ = 0.929, *P* = 0.405).  Figure 5(b) shows these results. We found no other interactive effect.

For amplitude, we found a significant interactive effect for load, word, and electrode site (*F*_(6,228)_ = 4.104, *P* = 0.005). In low-load tasks, a significant interactive effect was found for word and electrode site (*F*_(6,228)_ = 5.726, *P* = 0.001). We found a significant difference among words only in the partial sites (*F*_(3,114)_ = 9.958, *P* = 0.003), between pain and positive words (3.464 ± 0.528 *μ*V vs. 2.040 ± 0.490 *μ*V, *P* = 0.005), between pain and neutral words (3.464 ± 0.528 *μ*V vs. 1.380 ± 0.523 *μ*V, *P* = 0.001), and between pain and negative words (3.464 ± 0.528 *μ*V vs. 2.013 ± 0.513 *μ*V, *P* = 0.014).  Figure 5(e) shows these results. However, in high-load task, we found no significant interactive effect for word and electrode site (*F*_(6,228)_ = 0.776, *P* = 0.531) or main effects for word (*F*_(3,114)_ = 3.611, *P* = 0.065) and electrode site (*F*_(2, 76)_ = 0.189, *P* = 0.066). A significant difference occurred among electrode sites for each word (positive: *F*_(2, 76)_ = 7.798, *P* = 0.001; neutral: *F*_(2, 76)_ = 8.540, *P* = 0.004; negative: *F*_(2, 76)_ = 16.293, *P* < 0.001; and pain: *F*_(2, 76)_ = 23.277, *P* < 0.001), with the largest positive amplitude in the partial sites, followed by the central and frontal sites. We found no other interactive or main effects.

#### 3.5.3. N3

For latency, we found no interactive effects. A significant main effect was found for load (*F*_(1, 38)_ = 101.507, *P* = 0.002), with high-load tasks having longer latencies than low-load tasks (294.868 ± 4.637 ms vs. 280.926 ± 2.922 ms) did. We found a significant main effect for word (*F*_(3,114)_ = 4.449, *P* = 0.008), but only between positive words and neutral words (281.965 ± 2.999 ms vs. 292.057 ± 3.782 ms).  Figure 5(c) shows these results. We also found a significant main effect for electrode site (*F*_(2, 76)_ = 27.392, *P* < 0.001), with the partial sites (278.006 ± 3.099 ms) having the shortest latency, followed by the central (283.024 ± 3.562 ms) and frontal sites (302.662 ± 4.614 ms).

For amplitude, we found a significant interactive effect for load, word, and electrode site (*F*_(6,228)_ = 2.532, *P* = 0.049). In low-load tasks, a significant interactive effect was found for word and electrode site (*F*_(6,228)_ = 4.343, *P* = 0.007). We found significant differences among the words in the frontal (*F*_(3,114)_ = 10.506, *P* < 0.001), central (*F*_(3,114)_ = 15.653, *P* < 0.001), and partial sites (*F*_(3,114)_ = 14.242, *P* < 0.001), with the negative waves of neutral words being the longest.  Figure 5(f) shows these results.

We found no significant difference among different electrode sites for all words (positive: *F*_(2, 76)_ = 1.943, *P* = 0.171; neutral: *F*_(2, 76)_ = 3.497, *P* = 0.067; negative: *F*_(2, 76)_ = 3.962, *P* = 0.050; and pain: *F*_(2, 76)_ = 1.140, *P* = 0.296). However, in high-load tasks, no significant interactive effect occurred for word and electrode site (*F*_(6,228)_ = 2.758, *P* = 0.055). We found no other interactive or main effects.

## 4. Discussion

Many studies have examined the differences in the processing of different words on a behavioral level, but not sufficient for pain words for persons with pain. Our findings supported those of previous studies by reporting facilitative processing of special words [7, 22, 25] and added new evidence for a particular group of people.

Because behavioral performance in this study was the result of a combination of stimulus word interference and decision-making, the interfering effect of words cannot be judged only from the behavioral results. However, a significant load difference in both RT and AC supported that we properly applied the load factor in this experiment. In addition, the ERP results provided more detailed information for the neuronal processing of words between the EP and control groups.

### 4.1. AB to Pain Words in the EP Group Occurred Early

According to previous research focusing on emotional words or faces [7, 26], neural processing can be categorized into three stages. In stage 1, negative words or faces were distinguished early (N1 or P1), and neutral and emotional words or neutral and emotional faces were distinguished in stage 2 (VPP and N170), followed by stage 3, an in-depth assessment of the stimulation affective valence.

Our results were slightly different from those of previous studies in that both the control and EP groups did not show a preference for negative words. We deduced that this finding might be related to the warning nature of pain materials. Vigilance to pain is an environmental adaptation in human beings in order to warn them away from danger and is vital for human survival. In this way, pain imposes a higher priority on negative stimulus. Moreover, we found that neural processing of words is different between the groups. Semantic differentiation (between neutral and other words, N1) came first in the control group, followed by pain word identification (between pain and other words, P2). However, in the EP group, pain word identification occurred in the early stage (N1 and P2), and semantic differentiation followed (N3), suggesting an AB to pain.

AB described in the literature includes three characteristics [27]: attentional avoidance (e.g., allocating attention towards locations opposite to that of pain), facilitated attention (e.g., pain stimuli are detected faster or stronger than nonpain stimuli), and difficulty in disengagement (e.g., it is harder to disengage attention from a pain stimulus vs. a neutral stimulus). In our study, as compared with the control group, the EP group first showed attentional avoidance to pain words, revealed by N1 latency, with the longest latency in pain words as compared with positive, neutral, and negative words. Later, we found facilitated attention to pain words, revealed by P2 amplitude, with the highest amplitude in pain words as compared with other words, suggesting that processing pain materials occurred before processing the other words. Although we did not find difficulty in disengagement because of the short presentation times and the nontarget stimulus nature of the pain materials, these results validate our first hypothesis that AB to pain stimuli did happen at an early stage in the EP group.

N3, as a semantic differentiator in the EP group, was evident only in low-load tasks in stage 2. As Figure 5(f) shows, the amplitudes of neutral words had the largest negative waves, significantly different in every word type, which revealed a second stage of semantic processing in the brain. This finding was more evident for N1 (see Figure 3(d)) in both high- and low-load tasks for the control group.

### 4.2. Cognitive Load Is an Influencing Factor in Word Differentiation and AB

In the ERP results, we found that word differentiation mainly occurred in low-load tasks. In the control group, word differences in all N1, P2, and N3 latencies occurred only in low-load tasks (Figures 3(a)–3(c)), while in the EP group, word differences in P2 and N3 amplitudes occurred only in low-load tasks (Figures 5(e) and 5(f)). As cognitive load increased, participants were unable to distinguish potential word stimulus, and AB to pain vanished in the EP group, supporting our second hypothesis.

Cognitive load has long been researched for the interactive relation with attention and pain perception. According to cognitive load theory, human capacity of information processing is limited in that only a certain amount of information can be processed at a time. When a person engages in a variety of activities in performing difficult task, cognitive resources must be allocated to different tasks, which can tax the resources and drive down the efficiency of the task, called “cognitive overload.” Reduced pain perception is an effect of increased cognitive load in persons with pain. Legrain et al. [28] reported that cognitive load may help lower pain experiences by increasing distraction from pain. Some distraction paradigms also suggest that less pain is reported when performing a high-load task [20, 29]. fMRI researches [19–21] further supported this opinion by revealing that medium-to-high cognitive tasks, as compared with low cognitive tasks, can activate or deactivate brain areas related to pain.

In our study, however, we could not get concrete information about pain perception under high or low cognitive load because of the experiment protocol. It is hard to tell whether it is the result of alleviated pain perception under high cognitive tasks that led to no word differentiation or not. To our surprise, we discovered here that such phenomenon happened in both EP and control groups. As healthy subjects reported no pain in both high- and low-load tasks, pain relief resulting from higher cognitive task cannot provide a reasonable explanation. We thus suggested the notion of capacity limitations. As cognitive load theory raises, when the resources invested in cognitive tasks increased, the resources invested in other decreased. Word stimulus was designed as potential nontarget stimulus in our study. Therefore, when cognitive load increased, subjects had to pay more attention to complete the target cognitive tasks, while less attention was paid to nontarget word stimulus. As a result, word differentiation in the control group was insufficient, and AB to pain in the EP group disappeared under high-load tasks. We here for the first time suggested that cognitive load can influence word differentiation, as well as AB in pain subjects.

We must admit that experimental pain has fundamentally different qualities to clinical pain, in that the former is somewhat artificial, transient, and controllable. It would be intriguing to see, therefore, whether similar, or perhaps even stronger, attention-interference effects would be found in real world pains. Although our data are based on an experimental pain model, there are potential clinical implications if these results are replicated in real world pain, both acute pain and chronic pain, and shed light on AB management or intervention in the future pain treatment.

### 4.3. Electrode Effect Validates Attention Alerting to Pain Words

Prior studies have indicated a general dominance of the right hemisphere for all emotions [30, 31]. In our study, although not statistically treated, the amplitudes of all ERP components (N1, P2, and N3) elicited in the right hemisphere were greater than those elicited in the left hemisphere, a finding consistent with those of previous studies. In addition, we found electrode site effects among the frontal, central, and partial brain sites: N1 had the largest negative amplitude on the frontal sites in the control group, and P2 had the largest amplitude on the partial sites in both groups.

N1 amplitude in the control group peaked at the frontal sites, which is related to senior neural processing, such as planning, memorizing, and decision-making. Semantic differences were quickly identified in this region. P2 peaked at the partial brain regions, which may be highly related to attention alerting. As has been reported, the alerting network for attention is associated with areas in the parietal lobes, especially with the right hemisphere of the brain [32, 33]. Analysis of lateralization for patients with brain injury has indicated the right hemisphere's superiority to the alerting system [34], and the brain areas associated with innate vigilance are mainly in the parietal regions of this hemisphere [35]. In both the EP and control groups in our study, P2 was prominent in the partial sites, with a differentiation effect mainly for pain materials. Therefore, we propose that the electrode side effect on P2 was most likely related to both groups' attention alerting priority to pain words, which also is consistent with the warning nature of pain materials.

## 5. Study Limitations

Some limitations of this study call for further exploration in future research. First, we used a small sample size. However, despite this size, significant results emerged, which demonstrated attentional avoidance and the facilitated attention of pain words to other words in the EP group. Larger sample size may yield more findings. Second, the pain bias that we found may be affected by the participants' intensity of pain sensing and psychological traits (e.g., anxiety, depression, and pain catastrophizing). Further research should consider these issues to obtain more detailed information. Third, subjects in our study were participants with experimental pain, which is different from clinical pain patients. Further researches with real pain patients are suggested for future clinical application.

## 6. Conclusions

Our study provided additional evidence for AB to pain words in participants with experimental pain. The control group and EP group behaved differently with different words in neural processing. The EP group had an early pain bias, with a later somatic difference. Cognitive load is an influencing factor in word differentiation. It also affected AB in that as cognitive load increased, AB to pain disappeared. Future researches can be conducted on clinical pain in the hope for better treatment for pain.

## Figures and Tables

**Table 1:** Basic information for participants (*M* ± SD) [24].

		EP (*N* = 20)	CT (*N* = 20)	*t*, *X*^2^	*P*
Age (years)		22.54 ± 2.99	21.69 ± 1.89	0.745	0.397
Subject number (female)		20 (11)	20 (9)	0.133	0.715
Years of education		16.31 ± 2.25	15.77 ± 1.48	0.519	0.478
VAS value		5.38 ± 1.58	/	/	/
HADS	A	3.46 ± 2.40	3.08 ± 2.29	0.175	0.680
	D	4.00 ± 2.16	2.31 ± 2.14	4.033	0.056
STAI	S	35.76 ± 7.49	31.69 ± 8.04	1.792	0.193
	T	35.15 ± 7.35	31.78 ± 9.41	1.045	0.317

Abbreviations: EP: experimental pain persons; CT: control persons; VAS: visual analogue assessment scale; HADS: Hospital Anxiety and Depression Scale; STAI: State-Trait Anxiety Inventory; A: anxiety; D: depression; S: state of anxiety; T: trait of anxiety.

## Data Availability

The data used to support the findings of this study are available from the corresponding author upon request.

## Additional Points

Our manuscript's preprint can be found in the link at https://www.researchsquare.com/article/rs-66816/v1.
